# SNPs and Hox gene mapping in *Ciona intestinalis*

**DOI:** 10.1186/1471-2164-9-39

**Published:** 2008-01-25

**Authors:** Luigi Caputi, Marco Borra, Nikos Andreakis, Elio Biffali, Paolo Sordino

**Affiliations:** 1 Department of Biochemistry and Molecular Biology, Stazione Zoologica "A. Dohrn", Napoli, Villa Comunale, Italy; 2 Molecular Biology Service, Stazione Zoologica "A. Dohrn", Napoli, Villa Comunale, Italy

## Abstract

**Background:**

The tunicate *Ciona intestinalis *(Enterogona, Ascidiacea), a major model system for evolutionary and developmental genetics of chordates, harbours two cryptic species. To assess the degree of intra- and inter-specific genetic variability, we report the identification and analysis of *C. intestinalis *SNP (Single Nucleotide Polymorphism) markers. A SNP subset was used to determine the genetic distance between *Hox-5 *and *-10 *genes.

**Results:**

DNA fragments were amplified from 12 regions of *C. intestinalis *sp. A. In total, 128 SNPs and 32 one bp indels have been identified within 8 Kb DNA. SNPs in coding regions cause 4 synonymous and 12 non-synonymous substitutions. The highest SNP frequency was detected in the *Hox5 *and *Hox10 *intragenic regions. In *C. intestinalis*, these two genes have lost their archetypal topology within the cluster, such that *Hox10 *is located between *Hox4 *and *Hox5*. A subset of the above primers was used to perform successful amplification in *C. intestinalis *sp. B. In this cryptic species, 62 SNPs were identified within 3614 bp: 41 in non-coding and 21 in coding regions. The genetic distance of the *Hox-5 *and *-10 *loci, computed combining a classical backcross approach with the application of SNP markers, was found to be 8.4 cM (Haldane's function). Based on the physical distance, 1 cM corresponds to 39.5 Kb. Linkage disequilibrium between the aforementioned loci was calculated in the backcross generation.

**Conclusion:**

SNPs here described allow analysis and comparisons within and between *C. intestinalis *cryptic species. We provide the first reliable computation of genetic distance in this important model chordate. This latter result represents an important platform for future studies on Hox genes showing deviations from the archetypal topology.

## Background

A recent phylogeny placed Tunicata as the sister group of vertebrates [1]. This new position rejects traditional views of a Tunicata – Cephalochordata – Vertebrata succession [2-6] and it casts new light on comparative studies [7]. The taxonomic status of *C. intestinalis *L., the tunicate species most widely used for research purposes, was recently resolved with the discovery of two cryptic taxa, named *C. intestinalis *spp. A and B [8-11]. This finding prompts careful re-evaluation of research data, as it is reasonable that literature concerning *C. intestinalis sensu Linnaeus *refers to both cryptic species. Affinities with vertebrates are visible in the body plan organization of ascidian larvae and, despite major morphological rearrangements during metamorphosis, they are also retained in sessile adults [12,13]. Sequencing of *C. intestinalis *genome revealed an estimated number of protein-coding genes (15.852 over *ca*. 160 Mb genome length) similar to invertebrates and only about half of vertebrates [14]. Gene density is estimated to be 1 locus per 7.5 Kb (compared with 9 Kb in fruit fly and 100 Kb in human). *C. intestinalis *genes contain, on average, 6.8 exons. From the genomic point of view, the presence of several hundred genes having higher sequence similarity with *Drosophila melanogaster *and *Caenorhabditis elegans *than with vertebrates [14], as well as the small gene number, are indicative of species ancestry. Moreover, the genome is rich in AT (65%). Two derived features of the *C. intestinalis *genome are the presence of gene duplication events not detected in vertebrates, and the derived loss of ancestral genes that are conserved in chordates (*e.g*. paralogy of the Hox groups 7, 8 and 9) [14]. This latter phenomenon has been estimated to be around 35% and 45% more frequent in *C. intestinalis *than, respectively, in pufferfish and humans. Recent data on the congeneric species *C. savignyi *[15] revealed an impressive level of genomic variation, such that this species exhibits the "... highest rates of [...] polymorphisms ever comprehensively quantified in a multicellular organism". More specifically, *C. savignyi *shows a very high level of haplome-specific DNA (16.6%); this degree of variability between single haploid genomes originates from an enormous amount of various size indels throughout the genome [15].

Single nucleotide polymorphisms (SNPs) are one of the most important categories of genetic markers in the field of population genetics and human diseases. SNPs are base pair substitutions in the DNA of individuals [16], and are by far the most common type of molecular polymorphism in living organisms. Given this definition, single base pair insertions/deletions (indels) are not formally considered as SNPs. On the other hand, single base pair substitutions in cDNA are often included in this category of DNA variation, although they may result from errors in mRNA editing. About 1 SNP per Kb and 1 SNP per 125 bp occur in *Homo sapiens *[17] and *Aedes aegypti *[18], respectively. This very high variability represents a unique source of molecular markers. Biallelic SNPs are randomly distributed across the genome and have a low mutation rate (10^-8 ^– 10^-9^) [19]: this property makes it easier to calculate mutational rates in SNPs than, for example, in microsatellites. Although a restriction to four character states makes SNPs less informative than microsatellites for linkage and population genetics, synonymous coding as well as non-coding SNPs are still useful markers for these applications since they are not under natural selection. Non-synonymous SNPs in coding DNA regions are mostly used to enhance understanding of the molecular genetic basis of phenotypic variation, with a particular relevance for human-disease research. Technical progress in SNP detection [20] has turned this polymorphism into the most reliable marker for genomic approaches. In spite of their importance, few data concerning occurrence of SNPs in natural populations of lower chordates (cephalochordates and tunicates) are available [21]. Such analysis would greatly contribute to a deeper knowledge of genetic variability in evolutionary key model organisms, in particular when research is based on natural populations. In *C. intestinalis*, allelic polymorphism is equal to 1.2% on average, and it may reach peaks of 10–15% within short (100 bp) regions [14], although these data are still matter of debate [15]. Large-scale analysis in *C. savignyi *revealed an average SNP heterozygosity of 4.5%, with a Ts/Tv ratio of 2.45 and a *quasi-*equal distribution of the various types of transversions [15].

Genes belonging to the Hox family of transcription factors are control leaders in the definition of the antero-posterior axis of all bilaterians analyzed so far. Usually, Hox genes are structured in chromosomal clusters displaying an ordered succession of paralogy group members. A general dogma states the colinearity rule(s): more 3' located genes possess more anterior and earlier onsets of expression (spatial and temporal colinearity, respectively). Recent studies in tunicates [22,23] and in the sea urchin *Strongylocentrotus purpuratus *[24] revealed that coordinated spatial expression of Hox genes persists even in presence of a rearranged distribution of paralogy groups within the cluster. Therefore, understanding the evolutionary and functional scenarios of unclustered or unconventionally clustered Hox genes is a crucial task.

Herein, we studied SNP occurrence in *C. intestinalis *sp. A and sp. B. We developed exon-primed-intron-centered (EPIC) [25] primers in order to allow inter-specific genetic comparisons in 2 coding and 10 non-coding regions. For two adjacent Hox genes (*Hox-10 *and *-5*) displaying an inverted position, genetic distance and linkage disequilibrium (LD) were calculated using SNP markers in a backcross panel.

## Results and discussion

### DNA amplification and detection of SNPs

**Figure 1:**
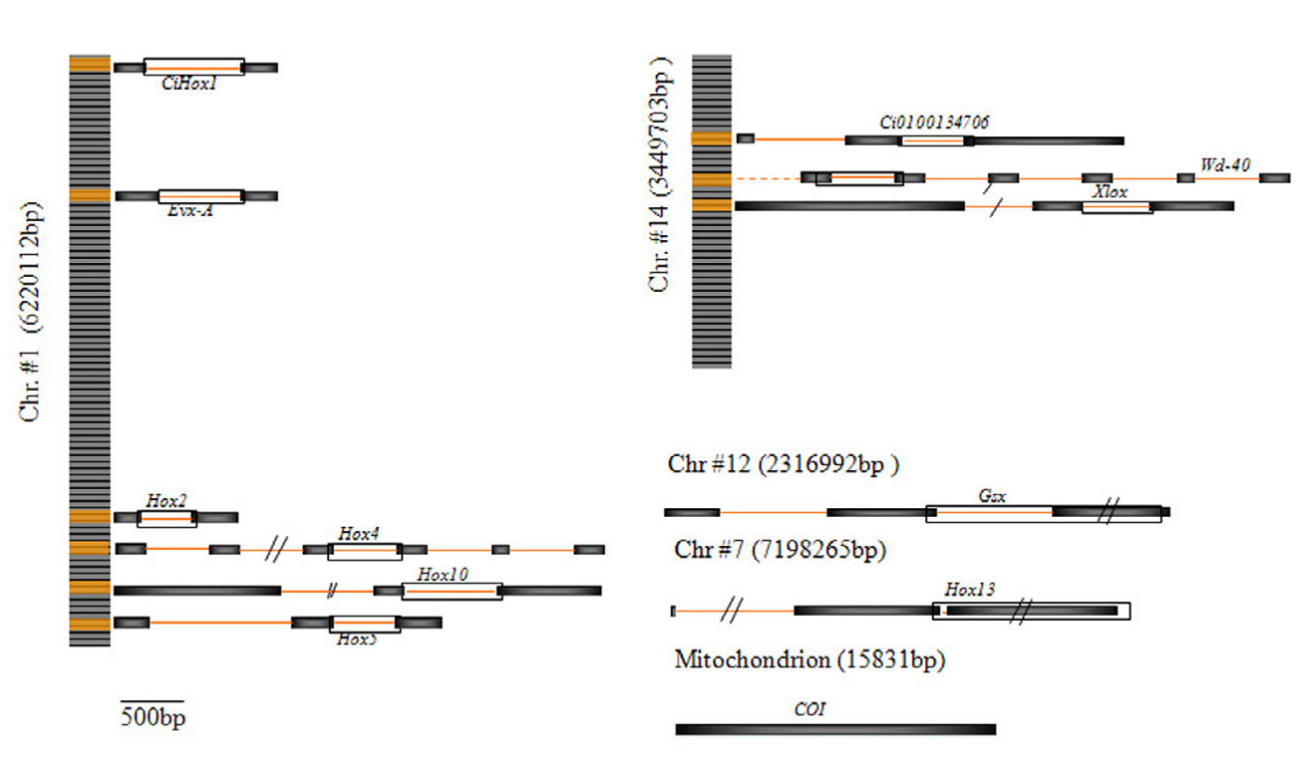
**Structure and location of loci**. Exon-intron structure and genomic location of genic loci used for analyses. Empty boxes: amplified regions. Chromosomes are numbered according to [28]. Distances between genes are indicative. All data are inferred from *C. intestinalis *sp. A.

A total of 16 EPIC and 2 non-EPIC primer pairs were PCR-tested on 30 genomic DNA samples from different *C. intestinalis *sp. A populations. Twelve primers were used to amplify reliable products (Fig. 1), ranging from 357 to 1340 bp length (Table 1). Consequently, we analyzed a total of 7966 bp: 5953 from intronic nuclear regions, 1498 from two nuclear exons (*Hox13 *and *Gsx*) and 515 from the mt-DNA *COI *(cytochrome oxydase subunit I) gene. We detected 128 transition/transversion SNPs (110 in non-coding regions) and 32 single base indels (29 in non-coding regions).

**Table 1:** Nucleotide polymorphisms *in C. intestinalis *sp. A loci.

**Gene**	**bp**	**nH**	**Ts**				**Tv**						**Ts/Tv**	**∑_SNPs(ts+tv)_**	**1 bp indel**	**∑_PS_**	**∑_SNPs_/_PS_**	**π**	**f_SNPs_**	**f_∑PS_**	**T93**	**L'sP**
			**AG**	**CT**	**∑_Ts_**	**%**	**AC**	**AT**	**GC**	**GT**	**∑_Tv_**	**%**										

*Hox1*	573	4	1	3	4	40	2	3	1	0	6	60	0.67	10	4	16	0.62	0.017	0.020	0.03	0.007	0.012
*Hox2*	531	3	1	0	1	50	0	1	0	0	1	50	0.5	2	1	3	0.67	0.002	0.004	0.005	0.000002	0.000066
*Hox4*	357	3	0	0	0	0	0	1	0	0	1	100	0	1	1	2	1	0.002	0.006	0.006	0.0028	0.0062
*Hox5*	481	11	10	8	18	72	1	6	0	0	7	28	2.57	25	3	45	0.55	0.024	0.052	0.093	0.004	0.004
*Hox10*	531	8	10	5	15	43	1	15	0	4	20	57	0.75	35	8	61	0.57	0.038	0.066	0.115	0.004	0.003
*EvxA*	569	3	0	0	0	0	0	2	0	0	2	100	0	2	1	3	0.67	0.002	0.003	0.005	0.0035	0.0039
*Xlox*	707	5	3	2	5	55	0	1	3	0	4	45	1.25	9	1	16	0.56	0.006	0.013	0.022	0.000002	0.0018
*Gsx*	186	6	1	1	2	100	0	0	0	0	0	0	0	2	0	15	0.13	0.012	0.01	0.08	0.0035	0.003706
*Wd-40*	1340	4	2	0	2	28	3	1	0	1	5	72	0.4	7	3	30	0.23	0.008	0.005	0.028	0.0084	0.231
*ci0100134706*	678	4	6	6	12	70	0	2	0	3	5	30	2.4	17	7	38	0.45	0.019	0.025	0.056	0.03	0.04
	**5953**		**34**	**25**	**59**	**54**	**7**	**32**	**4**	**8**	**51**	**46**	**1.18**	**110**	**29**	**229**	**0.48**		**0.019**	**0.038**		
																						
*Gsx*	897	4	5	8	13	87	0	2	0	0	2	13	6.5	15	0	15	1	0.011	0.017	0.017	0.00452	0.004575
*Hox13*	601	2	1	1	2	67	0	0	0	1	1	33	2	3	3	11	0.27	0.008	0.005	0.025	0.01188	0.016926
*COI*	515	1	0	0	0	0	0	0	0	0	0	0	0	0	0	0	0	0	0	0	0	0
	**2013**		**6**	**9**	**15**	**83**	**0**	**2**	**0**	**1**	**3**	**17**	**5.0**	**18**	**3**	**26**	**0.69**		**0.009**	**0.013**		

Abbreviations: bp, length of the amplified sequence; nH, number of haplotypes; Ts, transition; Tv, transversion; ∑, total; PS, polymorphic sites; π, nucleotide diversity; f, frequency; T93, Tamura-Nei genetic distance (1993); L'sP, Lake's paralinear genetic distance.

No SNPs were observed in the mitochondrial *COI *gene, 3 in the *Hox13 *and 15 in the *Gsx *coding regions. Our data indicate that the observed transition/transversion (T_s_/T_v_) ratio in the coding regions analyzed in this study is equal to 5.0 (Table 1). The T_s_/T_v _(1.37) observed within *C. intestinalis *sp. A coding and non-coding regions is considerably lower than the ratio found in *C. savignyi *(2.45) [15]. This difference is likely due to the regions here analyzed, namely non-coding intragenic and coding genic, whereas previous estimations were based on all genome sequences [15,16,26,27].

SNP frequency distribution in the non-coding DNA amplified regions displays a high degree of variability. *Hox-1*, *-2*, *-10, -5 *and *EvxA *genes are sequentially located along the chromosome #1 [23,28]. Notably, the highest SNP frequency occurs within those Hox genes (*Hox-5 *and *-10*) that have lost the archetypal genomic organization.

### Types and frequencies of SNPs

Overall, SNPs represent 48% of sequence polymorphisms in the 8 Kb region analyzed in this study, with a frequency of 1.61 SNPs every 100 bp (Table 1). The remaining 52% of polymorphisms consists of 1 bp or longer indels and multiple nucleotide polymorphisms. The high level of non-SNPs fits well with previous estimates in other multicellular organisms [15].

The occurrence of SNPs partially reflects the different *C. intestinalis *populations. For instance, all nine individuals from the Fusaro Lagoon display a T ↔ C transition at position 114 of the *Hox5 *locus, while only two individuals present an A ↔ G transition at position 111. In particular, most of non-synonymous changes are carried by individuals from geographically disjunct populations (*e.g*. California, Japan and Italy). Strain-specificity of non-synonymous SNPs is generally assumed to reflect adaptive responses to distinct environmental conditions [29-31].

**Figure 2:**
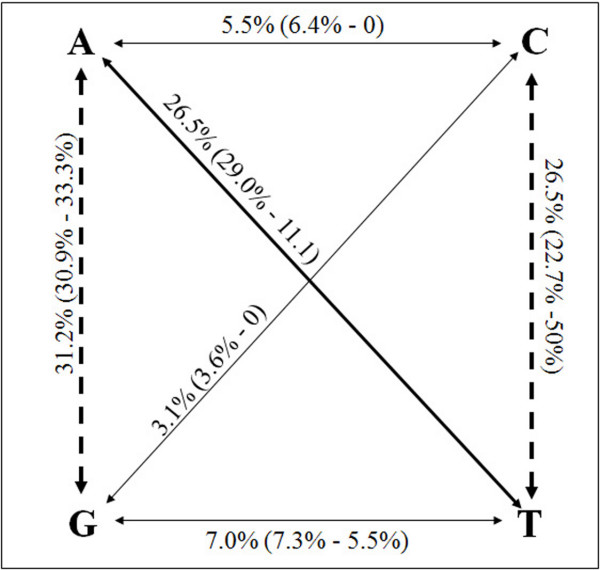
**SNPs in *Ciona intestinalis *sp. A**. Percentage of transversion and transition SNPs in *C. intestinalis *sp. A. Values outside parenthesis indicate the total percentage of each substitution type calculated over all DNA polymorphisms. Values within parenthesis indicate the percentage of each substitution type calculated, respectively, over non-coding and coding regions.

Concerning non-coding DNA regions, the overall number (∑_Ts _= 59; ∑_Tv _= 51 – Table 1) and frequency (f_Ts _= 0.01; f_Tv _= 0.008 – Table 1 and Fig. 2) of transition SNPs is slightly predominant. Furthermore, analysis of nuclear exonic regions indicates that the aforementioned transition prevalence is even more evident in the coding DNA (83% of total SNPs). This is not surprising since it is generally accepted that the frequently occurring 5-methylcytosine de-amination reactions cause transition overrepresentation in genomes [32]. Significant differences in the T_s_/T_v _ratio between non-coding and coding DNA regions have been previously observed [27], indicating that transitions occur more frequently in coding regions. Polymorphisms along the 1.5 Kb nuclear coding DNA causes 4 synonymous and 12 non-synonymous changes (Table 2). The synonymous/non-synonymous (S/nS) ratio (0.333) here reported is in contrast with the average S/nS ratio found in *C. savignyi *(5.16) [15]. It is assumed that overrepresentation of non-synonymous nucleotide changes is strictly associated with adaptive evolution [33,34]. In agreement, this mechanism may have played a crucial role in functional divergence of Hox genes after cluster duplications along the vertebrate lineage [35]. Being tunicates the closest relatives of vertebrates, our data lend support to the hypothesis that adaptive evolution on Hox and ParaHox genes was already active in basal chordates.

**Table 2:** *C. intestinalis *spp. A & B synonymous and non-synonymous substitutions.

**Gene**	**sp**	**S**	**nS**	**S/nS**
*Gsx*	A	1	8	0.12
*Hox13*	A	3	4	0.75
*COI*	A	0	0	-
*Hox13*	B	1	6	0.16
*COI*	B	2	5	0.40

Abbreviations: sp, cryptic species; S, synonymous substitutions; nS, non-synonymous substitutions.

Among different genes herein analyzed, non-coding DNA SNPs are highly variable in types and frequency. Transitions are predominant in 4/10 loci, while transversions prevail in 5/10. In one case (*Hox2*), T_s_/T_v _ratio is equal to one. The two transition substitution types are comparably represented (57.6% A ↔ G, 42.3% T ↔ C). Concerning transversions, A ↔ T substitutions are prevalent (70.6%), while A ↔ C, G ↔ C and G ↔ T are underrepresented (13.7%, 7.8%, 15.7%, respectively). However, 46.9% of all A ↔ T transversions are from a single intron (*Hox10*) and all four types of transversions are never observed at a single locus.

**Figure 3:**
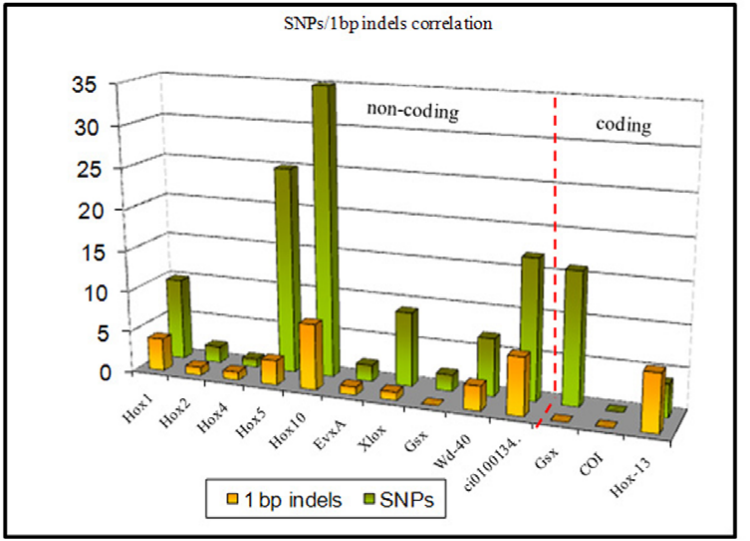
**Correlation between SNPs and 1 bp Indels distribution**. Quantitative relationships between SNPs and 1 bp indels in 13 genic loci of *C. intestinalis *sp. A. Except for *Gsx *and *Hox13 *coding sequences, the two types of polymorphism appear to be tightly correlated.

Twenty-nine 1 base-pair indels were found in the analyzed non-coding loci, and 3 in the *Hox13 *coding region. All indels in coding DNA were found in only one individual from an Adriatic sea population. Whether this data reflects partial or total loss of function of *Hox13 *remains elusive. Unlike coding sequences, all analyzed non-coding regions present a clear quantitative relationship between SNPs and 1 bp indels (Fig. 3), in support of some kind of structural and/or functional correlation between mechanisms leading to the appearance of different types of polymorphism [14].

### SNPs in *Ciona intestinalis *sp. B

All pairs of EPIC primers used to amplify *C. intestinalis *sp. A genomic DNA were successfully tested in *C. intestinalis *sp. B (Table 3). Among them, 5 associated with non-coding (*Hox-1*, *-2*, *-5*, *-10 *and *Xlox *- 2503 bp) and 2 with coding regions (*Hox13 *and *COI *- 1111 bp) have been used to investigate SNP presence, using 5 to 10 specimens for each locus. In total, 62 SNPs were detected (41 in non-coding, 21 in coding regions). On average, 1.89 SNPs were detected every 100 bp in coding regions, compared with 0.27 SNPs every 100 bp in the same *C. intestinalis *sp. A loci. In the cryptic species B, *Hox13 *SNP frequency is higher than the one detected in non-coding regions (f = 0.018 *vs *0.016). SNPs cause 1 synonymous and 6 non-synonymous amino acidic changes at the *Hox13 *locus, and 2 synonymous and 5 non-synonymous at the *COI *locus (Table 2). Previous analysis indicated the presence of a single mitochondrial haplotype shared by all Mediterranean populations of *C. intestinalis *sp. A. Conversely, *C. intestinalis *sp. B displays a variable COI in north European seas, suggestive of fixed populations [8]. Present results support the idea that the actual status of *C. intestinalis *sp. A in the Mediterranean Sea is due to a colonization by a mitochondrial variant, as well as the existence of more structured *C. intestinalis *sp. B populations [8].

**Table 3:** Nucleotide polymorphisms in *C. intestinalis *sp. B loci.

**Gene**	**bp**	**nH**	**Ts**				**Tv**						**Ts/Tv**	**∑_SNPs (*ts*+*tv*)_**	**1 bp indel**	**∑_PS_**	**∑_SNPs_/_PS_**	**π**	**f_SNPs_**	**f_∑PS_**	**T93**	**L'sP**
			**AG**	**CT**	**∑_Ts_**	**%**	**AC**	**AT**	**GC**	**GT**	**∑_Tv_**	**%**										

*Hox1*	349	2	0	1	1	100	0	0	0	0	0	0	-	1	0	1	-	0.002	0.003	0.003	0.0028	0.0028
*Hox2*	530	5	3	3	6	75	0	1	0	1	2	25	3	8	7	24	0.3	0.008	0.015	0.045	0.0019	0.0021
*Hox5*	383	5	4	2	6	37	4	3	0	3	10	63	0.6	16	1	27	0.4	0.028	0.042	0.07	0.0267	0.0268
*Hox10*	537	2	5	0	5	83	0	1	0	0	1	17	5	6	9	17	0.3	0.011	0.011	0.032	0.0116	0.0207
*Xlox*	704	9	4	3	7	70	0	1	2	0	3	30	2.3	10	11	35	0.3	0.006	0.014	0.05	0.000002	0.0017
	**2503**	**23**	**16**	**9**	**25**	**61**	**4**	**6**	**2**	**4**	**16**	**39**	**1.6**	**41**	**28**	**104**	**0.4**		**0.016**	**0.04**		
																						
*COI*	515	5	5	5	10	100	0	0	0	0	0	0	-	10	1	12	0.8	0.009	0.019	0.025	0.0039	0.0048
*Hox13*	596	5	6	3	9	82	0	1	0	1	2	18	4.05	11	1	23	0.5	0.016	0.018	0.038	0.0067	0.0099

**Tot**	**1111**		**11**	**8**	**19**	**90**	**0**	**1**	**0**	**1**	**2**	**10**	**9.5**	**21**	**2**	**35**	**0.6**		**0.019**	**0.031**		

Abbreviations: bp. length of the amplified sequence; nH. number of haplotypes; Ts. transition; Tv. transversion; ∑. total; PS. polymorphic sites; π. nucleotide diversity; f. frequency; T93. Tamura-Nei genetic distance (1993); L'sP. Lake's paralinear genetic distance.

Among SNPs in non-coding regions, the T_s_/T_v _ratio is equal to 1.56, and so it is similar to the ratio detected in sp. A (1.18). A ↔ G are more frequent than C ↔ T. A ↔ T are the most common transversion. The ratio of SNP mutations over total polymorphisms (∑_SNPs_/∑_PS_) is similar in the two cryptic species [∑_SNPs_/∑_PS _= 0.48 (sp. A) and ∑_SNPs_/∑_PS _= 0.39 (sp. B)].

In conclusion, all EPIC and non-EPIC primers that were designed taking advantage of the genome sequence of *C. intestinalis *sp. A, perfectly amplify homologous loci in *C. intestinalis *sp. B. Opposite to coding regions, frequency and T_s_/T_v _ratio in non-coding regions are very similar. Altogether, data are suggestive of genome behavior in the two cryptic species with shared and divergent traits.

### Genomic location

The organization of the Hox cluster in *C. intestinalis *[23] is characterized by an atypical arrangement. Two main differences can be observed: a) the cluster is broken (*Hox-12 *and *-13 *are located on a different chromosome) and b) paralogy groups do not respect the canonical 3' → 5' succession (*Hox-4 *and *-5 *are separated by *Hox10*) (Fig. 1). In this context, variability at these loci acquires a peculiar relevance. Our analysis shows (Table 1) that, among all analyzed loci (and among the same subset of specimens), *Hox5 *and *Hox10 *display the highest nucleotide diversity (π_*Hox5 *_= 0.0241; π_*Hox10 *_= 0.038) in non-coding regions. Similarly, the total number (∑_*Hox5 *_= 25; ∑_*Hox10 *_= 35) and frequency (f_*Hox5 *_= 0.052; f_*Hox10 *_= 0.066) of SNPs are the highest ones. In these loci, the number of observed transition substitutions is the highest among all genes analyzed, while the number of A ↔ T transversions is notably overrepresented in the *Hox10 *non-coding region (15 *vs *3.4 on average). Tamura and Nei (TN93) and Lake's Paralinear (L'sP) genetic distance calculated within the same sub-sample of specimens, concordantly assign higher values (TN93_*Hox5 *_= 0.004; TN93_*Hox10 *_= 0.004; L'sP_*Hox5 *_= 0.004; L'sP_*Hox10 *_= 0.003) to *Hox5 *and *Hox10 *than to any other loci screened for SNPs (Table 1).

The *Hox2 *and *Hox4 *genes have retained the archetypal genomic topology within the genome of *C. intestinalis *sp. A. These loci display a low SNP frequency (∑_*Hox-2,-4 *_= 3; f_*Hox-2,-4 *_= 0.0034). More generally, the total number of polymorphisms (including indels and multiple base polymorphisms – ∑_PS _= 5) and nucleotide diversity (∑_*Hox-2*,*Hox-4 *_= 0.0022) is notably low.

### Haplotypes structure, linkage disequilibrium and genetic mapping

**Figure 4:**
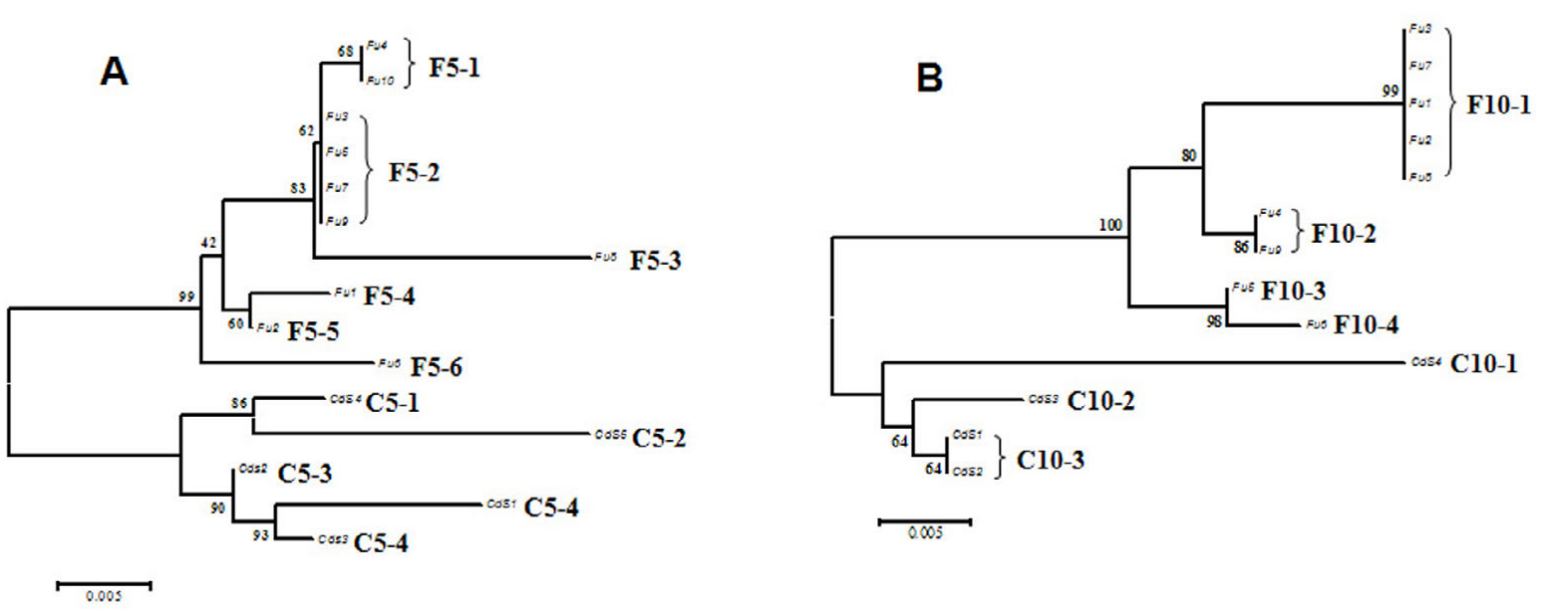
***Hox5 *and *Hox10 *haplotypes**. Maximum Parsimony trees of *Hox5 *(A) and *Hox10 *(B) haplotypes in Fusaro (F) and Castellamare di Stabia (C) populations. Individuals carrying the most common haplotypes (F5-1/C5-1 and F10-1/C10-3) were used as parents to generate the backcross progeny.

With the aim to genetically characterize populations of *C. intestinalis *sp. A inhabiting the Fusaro lagoon (FuI) and the harbour of Castellamare di Stabia (CdS) (Tyrrhenian Sea, Italy), we have identified 11 distinct haplotypes for the *Hox5 *locus, and 7 for the *Hox10 *one (Fig. 4). Specimens carrying the most frequent haplotypes (named F5-2, C5-1, F10-1, C10-3) have been selected as parental individuals in a backcross for SNP-based LD and genetic mapping of this region. In four (3.4%) of 120 backcross individuals, analysis failed to detect any SNPs in both loci. In addition, detection of *Hox5 *and *Hox10 *SNPs failed in, respectively, 6 (5.0%) and 4 (3.4%) individuals. The final matrix consisted of 116 samples, using a 50% threshold for missing genotypes. Observed *vs *expected heterozygosity was calculated with Haploview using phased haplotype files. We evaluated LD in the backcross generation using the Lewontin's D' [36] and correlation factor r^2 ^[37].

**Figure 5:**
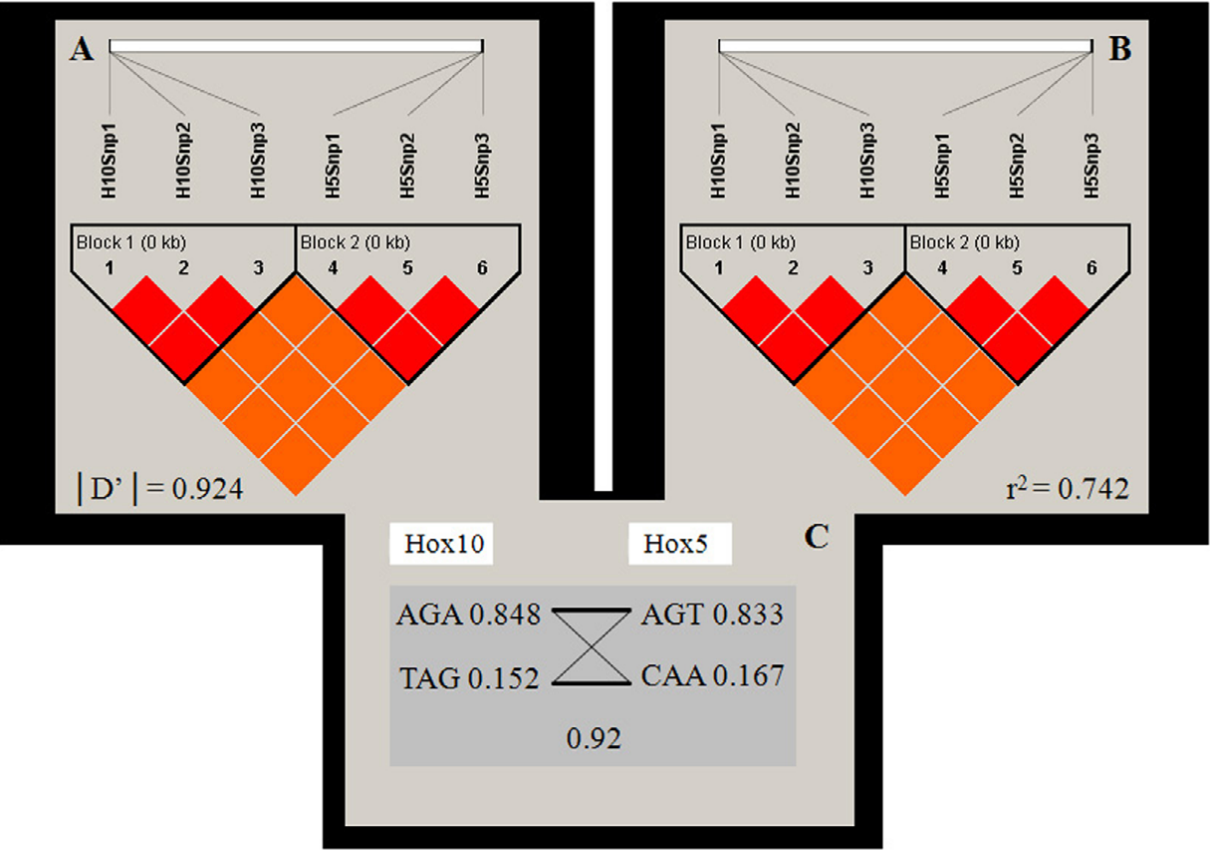
**Linkage disequilbrium**. Linkage disequilibrium |D'| (A) and r^2 ^(B) calculated between *Hox5 *and *Hox10 *loci using three SNP markers per locus. Values are only referred to orange blocks. (C) Haplotypes and their population frequency. Letter blocks correspond to the six SNP types. Thin lines connect haplotypes with a frequency > 0.1%; thick lines connect haplotypes with a frequency > 10.0%. The recombination D' value between the two blocks is shown.

**Figure 6:**
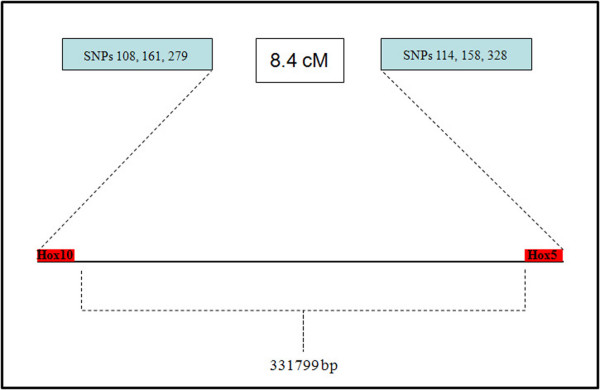
**Genetic and physical map**. Genetic and physical distances between *Hox10 *and *Hox5 *loci computed using 3 SNP markers per gene in the backcross generation. Numbers following SNPs indicate the substitution position within the sequence. Physical distance was inferred from the JGI *C. intestinalis *v.2.0 genome sequence.

Linkage disequilibrium [D'] between genic loci is equal to 0.92, and r^2 ^is 0.74 (Fig. 5). The coefficient r^2 ^increases in the backcross generation from the value detected in F_0 _(0.67). A genetic map was generated anchoring SNP loci to the same chromosome with MapMaker/exp v.3. The obtained cM value (according to Haldane's function) is 8.4, with a 28.59 threshold between the two loci. Linkage was correlated with physical distances by using a genome browser (*C. intestinalis *v2.0, Joint Genome Institute) [14]. Being *Hox10 *and *Hox5 *separated by 331799 Kb, including some unresolved nucleotide stretches (N), 1 cM corresponds to 39.5 Kb (Fig. 6).

The recombination ratio for the *Hox10*/*Hox5 *region in *C. intestinalis *sp. A is consistent with previous results, despite some ambiguity concerning the taxonomic status of parents [38]. In the highly polymorphic Fu/Hc locus of the colonial tunicate, *Botryllus schlosseri*, 1 cM corresponds to approximately 90 Kb [39]. Hence, the high recombination rate of the *Hox10*/*Hox5 *region is suggestive of a peculiar variability, and it provides an interesting point of discussion about disrupted topology of Hox clusters.

### SNP reliability

Different methods were used in order to assess the validity of the SNPs analyzed in the present paper. First, only SNPs confirmed by two independent PCR, three clones for each PCR, were kept for further analysis. This first step was applied in both *C. intestinalis *sp. A and sp. B. Second, we analyzed segregation ratios in the FuI/CdS F_1 _crosses. All SNPs displayed the expected 1:1 ratio of Mendelian inheritance. Therefore, all data herein reported are reliably not due to sequencing artifacts.

## Conclusion

We identified 128 SNPs through sequencing of 8 Kb genomic DNA of *C. intestinalis *sp. A. All primers used to amplify genomic DNA were successfully tested in *C. intestinalis *sp. B, allowing inter-specific comparison. As expected, SNP frequency is lower in coding than in non-coding regions [40,41]. Also, SNP frequency is not constant in intronic DNA. Variability likely depends on the genomic location of analyzed sequence. In particular, we identified a highly polymorphic region in correspondence of *Hox5 *and *Hox10*, two genes that have inverted their paralogy group position within the typical topology of Hox clusters. The dominance of non-synonymous *vs *synonymous SNPs in Hox coding regions suggests that adaptive evolution is acting on these genes. In order to establish the genetic map of this region, we performed a SNP-based approach to measure cM distance between two Hox genes in a backcross generation. We calculated linkage disequilibrium and correlated genetic and physical distances.

In this report, we analyzed SNP occurrence in *C. intestinalis *sp. A at intra- and inter-population levels, providing an important source of genetic markers for linkage, population and comparative studies. Our data indeed represent a further step toward the establishment of a unique integrative system for comparative genomics in chordates, consisting of two cryptic (*C. intestinalis *sp. A & B) and one congeneric (*C. savignyi*) species.

We also calculated the genetic distance within a genomic region of particular interest. This data will contribute to in-depth investigations concerning the mechanisms the maintain Hox colinearity in absence of a coordinated genomic organization.

## Methods

### Collection of *Ciona intestinalis *sp. A and B specimens

Individuals of *Ciona intestinalis *sp. A were collected in the following localities: Fusaro Lagoon (Italy), Castellamare di Stabia (Italy), Villaggio Coppola (Italy), Taranto (Italy), Venice (Italy), Lake Timsah (Egypt) and Alicante (Spain). Californian and Japanese sequences were obtained from the JGI *Ciona intestinalis *v2.0 genome [42] and the Ghost Database [43].

*Ciona intestinalis *sp. B specimens were obtained from the following localities: Plymouth Sound and Edinburgh (United Kingdom), Brest (France), Breskens Harbour (The Netherlands) and Fiskebäckskil (Sweden).

### DNA amplification and sequencing

Genomic DNA was extracted as previously described [8]. Amplification of DNA fragments was performed as in [8], except for the following loci: *Hox-1*, *-2*, *-10*, *EvxA*, *Xlox*, *Wd-40 *and *ci0100134706 *(see Table 4 for details). PCR products were extracted and purified using the QIAquick Gel Extraction Kit (Qiagen), and then cloned into TOPO TA Cloning Vector (Invitrogen) following manufacturer's instruction. Two independent PCR and three clones for each gene were sequenced using the Applied Biosystems 3730 DNA Analyzer Apparatus at the Molecular Biology Service (SBM) of the Stazione Zoologica "A. Dohrn" in Naples. Sequences were automatically aligned using ClustalW [44] and hand-checked with Bioedit v. 7.0.5.3 [45].

**Table 4:** Markers, primers and thermal cycle conditions.

**Marker**	**Forward oligo (5' to 3')**	**Reverse oligo (5' to 3')**	**Cycle conditions**
*Hox1*	GCATTGGGCCTTAATGAAACCC	CTTCTGCTTCATACGTCGAT	95°C (3'). [94 (30"). 56°C. (30"). 72°C(1')]x34. 72°C. (3')
*Hox2*	CGGACTGCTTACACCAACACC	TCGGCGCTTGTTACGTCACA	95°C (3'). [94 (30"). 55°C. (30"). 72°C(1')]x30. 72°C. (3')
*Hox4*	ACGCGACACCAGGTACTTGAA	ATATGCACGGCCGTGGGAAA	95°C (3'). [94 (30"). 57°C. (30"). 72°C(1')]x30. 72°C. (3')
*Hox10*	GCAAGAAACGAGTGCCGTACA	CTTCACTTGACGGTCGGTAAG	95°C (3'). [94 (30"). 57°C. (30"). 72°C(1')]x30. 72°C. (3')
*Wd-40*	TAGCTCGAGTTTGGGATATG	TGGGTTAAGAGGGTGAGTGG	95°C (5'). [94 (1'). 54°C. (2'). 72°C(3')]x35. 72°C. (10'). 72°C. (3')
*0100134706A*	TGTTCAGACCAGCATTACTGGC	GAGATCGCATTACGGACATTG	95°C (3'). [94 (30"). 53°C. (30"). 72°C(1')]x30. 72°C. (3')
*EvxA*	GGCCAACGTGCGTCGTTAT	ACGGCCACGTCTGCCGTTGT	95°C (3'). [94 (30"). 55°C. (30"). 72°C(1')]x30. 72°C. (3')

### SNP discovery and analysis

SNPs were identified as sequence differences in the alignment. Only SNPs detected in all different trials were considered valid. All analyses were performed using DAMBE v.4.5.33 [46] and DnaSP v.4.0 [47]. Maximum Parsimony trees were inferred using MEGA v.3.1 [48] with 1000 bootstrap replications. To facilitate detection of SNPs for backcross genotyping, we have chosen 3 SNPs for each of the two Hox genes. Marker selection was done considering a) distance between SNPs (primer design), b) character polymorphism (detection of base changes), and c) F_1 _inheritance (transmission probability). According to these criteria, SNPs in position 114, 158, 328 were selected for *Hox5*, and those in position 108, 161, 279 for *Hox10 *(Table 5).

**Table 5:** SNP primers and FuI *vs *CdS polymorphic sites.

**Primer**	**Sequence (5' to 3')**	**Fu *vs* CdS SNP**
5SNP114	(GACA)_2_GATGTTTATGACGAAGAA	A – C
5SNP158	GACACGAGTTGTTTGGGTAATGG	G – A
5SNP328	CAGATATTGGACCAAAAGTTCC	T – A
10SNP108	(GACA)_3_TTATAATATATCTCTTGT	A – T
10SNP161	CAGATTTTATTTTTGTGAATTA	G – A
10SNP279	GACACAAATACTTGATTAAGTA	A – G

### Culturing

Fusaro/Castellamare di Stabia hybrids were cultured according to a published protocol [49], with modifications.

### Linkage disequilibrium and Genetic mapping

SNP oligos used to perform the backcross analysis are described in Table 5. Sample reactions were prepared in 10 μl containing 5 μl of SNaPshot Multiplex Ready Reaction Mix (Applied Biosystems, Foster City, CA., USA), 3 μl of PCR products, 1 μl of specific primers, 1 μl of deionized water. Thermal cycling was performed on a MJ DNA Engine PTC 200 at SBM, following standard procedure using an annealing temperature of 42°C. Post-extension treatment was performed using the Applied Biosystems 3730 DNA Analyzer. Data were analyzed by eye with GeneMapper v.3.7 (Applied Biosystems).

The metric D is a quantitative measure of allelic association. Given the two sites 1 and 2, *x*_12 _is the frequency of the corresponding haplotype and *p*_1_, *q*_2 _are the marginal allele frequencies. Hence, D = *x*_12 _– (*p*_1_)(*q*_2_) [50]. D' is obtained by normalising D over the theoretical maximum D, given the specific allele frequencies, such that D' = D/D_max _[36] Finally, the correlation factor r^2 ^is equal to D^2^/(*p*_1_)(*q*_2_).

Normalized linkage disequilibrium measure [D'] and the correlation coefficient (r^2^) were calculated and visualized using HaploView v.3.32 [51] with a phased genotype matrix. Genetic distance (cM) between *Hox-5 *and *-10 *loci was calculated using MapMaker/EXP v.3.0 [52] with a threshold (LOD) greater than 3 and ordered with a LOD score of 1.44 [38]. The genetic distance was calculated using Haldane's mapping function [53].

## Authors' contributions

LC (corresponding author) performed acquisition and analysis of data and drafted the manuscript; MB and EB developed the SNP detection procedure; NA gave a fundamental help in revisiting and improving the quality of the manuscript; PS is the PI who conceived and coordinated the project, and made important contributions to the text. All authors read and approved the final manuscript.
